# Hallazgos incidentales de COVID-19 en PET/CT 18F-FDG de pacientes asintomáticos con cáncer en dos instituciones de salud de Bogotá, Colombia

**DOI:** 10.7705/biomedica.5833

**Published:** 2020-11-12

**Authors:** Alejandro Martí, Sarai Morón, Eliana González, Julián Rojas

**Affiliations:** 1 Unidad de PET/CT, Instituto de Diagnóstico Médico, Idime, Bogotá, D.C., Colombia Idime BogotáD.C Colombia; 2 Medicina Nuclear y Unidad PET/CT, Instituto Nacional de Cancerología, Bogotá, D.C., Colombia Instituto Nacional de Cancerología BogotáD.C Colombia

**Keywords:** infecciones por coronavirus, cáncer, medicina nuclear, tomografía de emisión de positrones, reacción en cadena de la polimerasa de transcriptasa inversa, Coronavirus infections, cancer, nuclear medicine, positron-emission tomography, reverse transcriptase polymerase chain reaction

## Abstract

La COVID-19 es la infección viral causada por el SARS-CoV-2 y declarada por la Organización Mundial de la Salud (OMS) como pandemia. Los pacientes con cáncer tienen un mayor riesgo de adquirir la infección y un peor pronóstico, ya que deben asistir a visitas médicas en diferentes centros hospitalarios, reciben tratamientos médicos y quirúrgicos y deben someterse a estudios diagnósticos como la PET/CT en servicios de medicina nuclear, lo que es ocasión para el hallazgo incidental de la infección. Se presentan las imágenes de tomografías computarizadas por emisión de positrones con 18-fluorodesoxiglucosa (F18) *(Positron Emission Tomography and Computed Tomography with 2-deoxy-2-[fluorine-18]fluoro-D-glucose,* PET/CT F18-FDG) en las que se evidenció la COVID-19 en pacientes con diversas enfermedades oncológicas, pero sin sintomatología respiratoria.

## Abstract (en)

COVID-19 is the viral infection caused by SARS-CoV-2 declared by the World Health Organization (WHO) as a pandemic. Patients with cancer have a higher risk to acquire the infection and worse prognosis as they have to attend more medical visits in healthcare institutions, receive medical and surgical treatments, and be subjected to diagnostic studies such as PET/CT in nuclear medicine services where the infection may be an incidental finding. We present here F18-FDG PET/CT *(Positron Emission Tomography and Computed Tomography with 2-deoxy-2-[fluorine-18]fluoro-D-glucose),* images with findings of COVID-19 from patients with different oncological conditions but no respiratory symptoms.

El 30 de enero de 2020, el Director General de la Organización Mundial de la Salud (OMS) declaró el brote de enfermedad por coronavirus (COVID-19), notificado por primera vez en Wuhan (China) el 31 de diciembre de 2019, como una emergencia de salud pública internacional ^1^. La COVID-19 es la infección viral causada por el SARS-CoV-2 que, para septiembre del 2020, ya había contagiado a más de 25 millones de personas en el mundo y causado la muerte de más de 840.000 ^2^. El 28 de septiembre de 2020 los casos confirmados en Colombia alcanzaron los 818.203 y las muertes ya eran 25.641 ^3^. En estos momentos, el diagnóstico de la enfermedad es principalmente clínico y se confirma mediante una prueba de reacción en cadena de la polimerasa (PCR).

Las unidades de imágenes diagnósticas y medicina nuclear no han sido ajenas a la enfermedad. Como ha sucedido con el resto de especialidades, los pacientes han continuado asistiendo a practicarse los exámenes diagnósticos y los procedimientos terapéuticos que requieren ^4^, lo cual implica la posibilidad de registrar hallazgos incidentales en las imágenes de medicina nuclear, como la tomografía PET/CT F18-FDG *(Positron Emission Tomography and Computed Tomography with 2-deoxy-2-[fluorine-18]fluoro-D-glucose)* en pacientes asintomáticos sin diagnóstico previo de COVID-19.

Se ha reportado una prevalência de hallazgos incidentales de casos sospechosos de COVID-19 de hasta el 16,25 % en este tipo de exploraciones ^5^, los cuales incluyen opacidades pulmonares con patrón de vidrio esmerilado, engrasamiento de los tabiques interlobulillares e intralobulillares, opacidades alveolares (consolidaciones), engrosamientos broncovasculares y bronquiectasias por tracción. Es común el compromiso bilateral y multilobar, sobre todo en las zonas inferiores. Estas alteraciones se acompañan de un incremento metabólico del trazador, con rangos en el *Standardized Uptake Value* (SUVmáx) de 3,7 a 12 ^6^. Asimismo, se debe tener en cuenta que los pacientes con cáncer tienen un mayor riesgo de infección por COVID-19. Cerca del 40 % de los pacientes con cáncer y COVID-19 han debido ser hospitalizados, el 20 % desarrolló la enfermedad respiratoria grave y el 12 % murió en el curso de los 30 días posteriores al diagnóstico ^7^.

Además, este tipo de pacientes evolucionan, al parecer, con peor pronóstico porque deben asistir a más citas médicas en diferentes centros hospitalarios para las quimioterapias, los procedimientos médicos y quirúrgicos, y los frecuentes estudios diagnósticos en servicios de medicina nuclear, radiología y PET/CT. Suelen llegar asintomáticos y, por ello, los médicos especialistas en medicina nuclear y los radiólogos deben estar atentos a dichas eventualidades.

Se presentan aquí imágenes de PET/CT F18-FDG con hallazgos incidentales de COVID-19 de pacientes con diferentes condiciones oncológicas, pero sin sintomatologia respiratoria. Los pacientes ingresaron al servicio de medicina nuclear y PET/CT de dos instituciones de salud de Bogotá y, debido a los hallazgos de las imágenes, se remitieron para la prueba de PCR, la cual fue positiva.

## Caso 1

**Figura 1:**
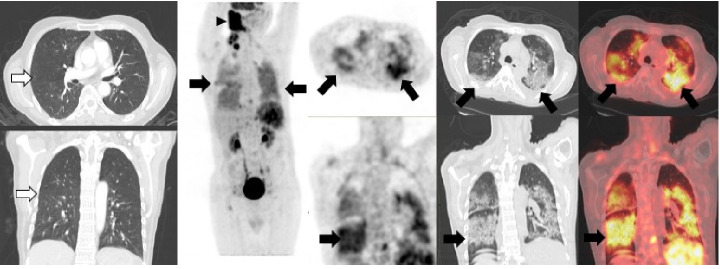
Tomografia de tórax de alta resolución previa a la PET/CT F18-FDG en la que no se evidenció ningún tipo de alteración parenquimatosa (flechas blancas). En la PET/CT F18-FDG se evidenció una masa hipermetabólica intensa, SUVmáx de 14, en la región cervical derecha y áreas de ulceración cutánea, conglomerados adenopáticos supraclaviculares derechos y múltiples focos hipermetabólicos infiltrativos en el parénquima esplénico relacionados con compromiso por linfoma (cabeza de flechas negras). En el tórax (flechas negras) se detectaron derrames pleurales bilaterales sin actividad metabólica y múltiples opacidades alveolares hipermetabólicas, así como un patrón de vidrio esmerilado de distribución en parches de carácter multilobar en ambos parénquimas pulmonares, con SUVmáx de 4,5.

Hombre de 60 años, asintomático respiratorio, con antecedentes de linfoma B difuso de células grandes, remitido para PET/CT F18-FDG de estadificación inicial (figura 1). Antes de este diagnóstico, el paciente se había hecho una tomografia de tórax el 14 de julio de 2020 en la cual no se evidenciaba ninguna alteración. El 4 de agosto siguiente, se le tomó una PET/CT con F18-FDG en el Instituto Nacional de Cancerología de Bogotá, y se encontró una masa hipermetabólica en la región cervical derecha, conglomerados adenopáticos supraclaviculares derechos y focos hipermetabólicos en el parénquima esplénico relacionados con compromiso por linfoma. En el tórax se detectaron derrames pleurales bilaterales y múltiples opacidades alveolares hipermetabólicas. Dados estos hallazgos, se remitió al paciente para que se le practicara una prueba de PCR para diagnóstico de la COVID-19, la cual fue positiva.

## Caso 2

**Figura 2:**
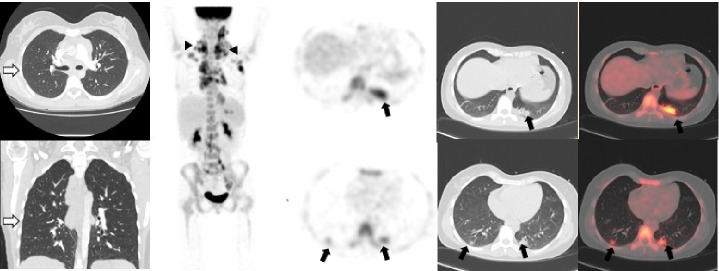
Angiotomografía de tórax previa a la PET/CT en la que no hubo evidencia de compromiso parenquimatoso (flechas blancas). En la PET/CT F18-FDG se encontraron múltiples ganglios hipermetabólicos cervicales, supraclaviculares, axilares bilaterales y mediastinales con SUVmáx de 10,7, relacionados con la enfermedad de base (cabezas de flechas). En el tórax (flechas negras) se observaron opacidades parenquimatosas con patrón de vidrio esmerilado localizadas en ambos segmentos posteriores de los lóbulos inferiores, pero con mayor compromiso del lado izquierdo, con aumento de la actividad metabólica y SUVmáx de 5,6.

Mujer de 26 años, sin sintomatología respiratoria, con diagnóstico de linfoma B difuso de células grandes, remitida para PET/CT F18-FDG de estadificación inicial (figura 2). El 7 de agosto de 2020 se le tomó una angiotomografía de tórax en la que no se observaba compromiso parenquimatoso (flechas blancas). Posteriormente, el 13 de agosto, se le tomo una PET/CT F18-FDG en el Instituto Nacional de Cancerología de Bogotá, y se evidenció compromiso ganglionar hipermetabólico cervical, supraclavicular, axilar bilateral y mediastinal relacionado con su condición de base. Incidentalmente se observaron en el tórax opacidades parenquimatosas con patrón de vidrio esmerilado que no se evidenciaban en la angiotomografía computarizada previa. Dada la pandemia actual, se envió a la paciente para que se le practicara una prueba de PCR para diagnóstico de la COVID-19, con resultado positivo.

## Caso 3

**Figura 3:**
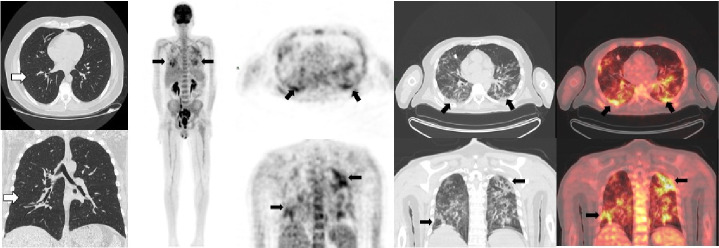
Tomografía computarizada de alta resolución de tórax sin presencia de compromiso parenquimatoso (flechas blancas). En la PET/CT F18-FDG se observó en el segmento del tórax múltiples opacidades con patrón de vidrio esmerilado, dispersas en forma de parches en ambos parénquimas pulmonares basales derechas, dominantes, con elevada actividad metabólica y SUVmáx de 7 (flechas negras).

Hombre de 53 años con linfoma b difuso transformado de folicular sometido a múltiples esquemas de quimioterapia. Se le hizo la PET/CT F18-FDG para la evaluación final del tratamiento (figura 3). Se le había hecho una tomografía computarizada de alta resolución de tórax el 29 de agosto de 2020 y no presentaba compromiso parenquimatoso. La PET/CT F18-FDG se hizo el 13 de agosto en el Instituto Nacional de Cancerología de Bogotá, y no se evidenciaron adenopatías o conglomerados hipermetabólicos infiltrativos asociados con la enfermedad de base del paciente. Sin embargo, en el segmento del tórax se evidenciaron múltiples opacidades con patrón de vidrio esmerilado y dispersas en ambos parénquimas pulmonares con gran actividad metabólica. Dados estos hallazgos se envió al paciente a la toma de la PCR para COVID-19, la cual resultó positiva.

## Caso 4

**Figura 4:**
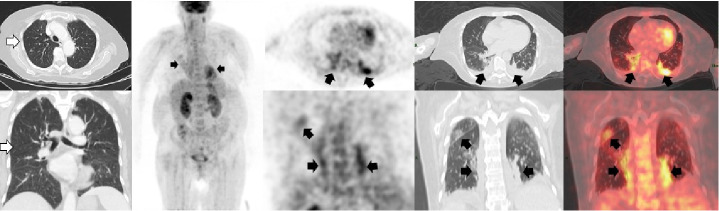
Tomografía computarizada de alta resolución de tórax (flechas blancas) en la que se observaron cambios fibroatelectásicos y bronquiectasias por tracción en el lóbulo superior derecho. En la PET/CT F18-FDG (flechas negras) no hubo evidencia de lesiones hipermetabólicas sospechosas de compromiso secundario. Sin embargo, en el tórax se observaron múltiples opacidades con patrón de vidrio esmerilado y aumento difuso del trazador y SUVmáx de 4, localizadas en ambos campos pulmonares con una consolidación hipermetabólica del segmento posterior del lóbulo inferior izquierdo y derrame pleural bilateral sin actividad metabólica.

Mujer de 57 años sin síntomas respiratorios y con antecedentes de linfoma no Hodgkin de células B de la zona marginal. Se solicitó la PET/CT F18-FDG para valorar la respuesta al final del tratamiento (figura 4). El 17 de agosto de 2020 se le había hecho una tomografía computarizada de alta resolución de tórax en la que solo se detectaron algunas atelectasias en el lóbulo superior derecho. En la PET/CT realizada el 25 de junio de 2020 en el Instituto Nacional de Cancerología, no se evidenciaron lesiones hipermetabólicas sospechosas de compromiso secundario. En el tórax se observaron múltiples opacidades en vidrio esmerilado con aumento difuso del trazador asociado con una consolidación hipermetabólica y derrame pleural bilateral sin actividad metabólica. Con estos hallazgos la paciente fue remitida para que se le practicara una prueba de PCR para COVID-19 y el resultado fue positivo.

## Caso 5

**Figura 5:**
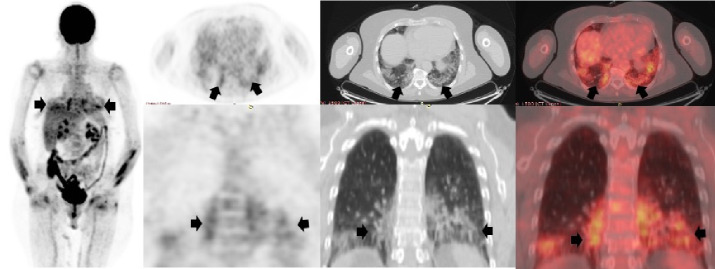
PET/CT con F18-FDG en la que no se observaron lesiones hipermetabólicas relacionadas con la enfermedad de base. Sin embargo, en el tórax se evidenciaron múltiples opacidades en parches con patrón de vidrio esmerilado y actividad metabólica difusa en el lóbulo medio, la língula y en ambos lóbulos inferiores, con SUVmáx de 3,6 (flechas negras), y de aspecto inflamatorio e infeccioso sospechosos de COVID-19.

Mujer de 74 años con antecedentes de linfoma no Hodgkin folicular de grado histológico 2, quien recibió seis ciclos de quimioterapia. Se hizo la PET/CT F18-FDG para valorar la respuesta al tratamiento en el Instituto de Diagnóstico Médico (Idime) en Bogotá (figura 5). Los hallazgos de la PET/CT fueron negativos para lesiones hipermetabólicas relacionados con la enfermedad de base. En el tórax se evidenciaron opacidades con patrón de vidrio esmerilado y actividad metabólica difusa de aspecto inflamatorio e infeccioso. Dada la situación actual de pandemia, se hizo el seguimiento clínico de la paciente para descartar la COVID-19 y el resultado de la PCR fue positivo.

## Caso 6

**Figura 6:**
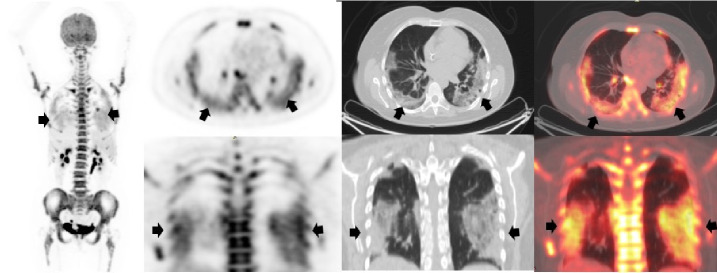
PET/CT F18-FDG en la cual se observó engrosamiento difuso con actividad metabólica irregular y SUVmáx de 3,7 en la pared gástrica de la topografía del antro y del cuerpo y el antro relacionada con el compromiso infiltrativo por la condición primaria conocida. En el segmento pulmonar (flechas negras) se evidenciaron múltiples opacidades con patrón de vidrio esmerilado, hipermetabólicas y dispersas en ambos parénquimas pulmonares, de localización periférica, SUVmáx de 6,5, de aspecto inflamatorio e infeccioso y sospechosas de COVID-19.

Mujer de 68 años, sin síntomas respiratorios previos, con antecedentes de carcinoma gástrico de patrón difuso de células en anillo de sello, etapa IV, con compromiso peritoneal, tratada con ocho ciclos de quimioterapia, el último de ellos el 10 de agosto de 2020. La PET/CT F18-FDG (figura 6) se hizo para valorar el tratamiento en el Instituto de Diagnóstico Médico (Idime) de Bogotá. En la PET/CT se detectó engrosamiento difuso de la pared gástrica relacionado con el compromiso infiltrativo causado por la condición primaria. En el segmento pulmonar se observaron opacidades con patrón de vidrio esmerilado hipermetabólicas, de aspecto inflamatorio e infeccioso, en ambos parénquimas pulmonares. Se remitió a la paciente para la toma de la PCR de diagnóstico de la COVID-19, la cual fue positiva.

## Caso 7

**Figura 7:**
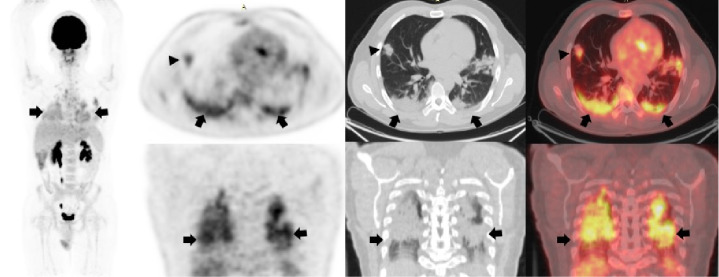
PET/CT F18-FDG que evidenció en el tórax (flechas negras) múltiples opacidades nodulares y pseudonodulares en ambos parénquimas pulmonares, así como opacidades alveolares con broncograma aéreo, multilobares en ambas bases, algunas dispersas en los lóbulos superiores y la língula, con SUVmáx de 3,8 dominante en la base derecha, y opacidades nodulares mal definidas, con broncograma, dispersas aleatoriamente, y nódulo irregular hipermetabólico en el segmento lateral del lóbulo medio (cabeza de flecha) de 24 mm de diámetro y densidad de partes blandas, con SUVmáx de 3,3.

Hombre de 34 años, sin síntomas respiratorios previos, con antecedentes de seminoma tratado con orquiectomía izquierda y cuatro ciclos de quimioterapia, el último de ellos el 29 de abril de 2020. Se lo remitió para tomarle una PET/ CT F18-FDG (figura 7) al instituto Idime para nueva estadificación y en ella se observaron en el tórax múltiples opacidades nodulares en ambos parénquimas pulmonares altamente sospechosos de COVID-19, por lo que se le envió a para que se le practicara una prueba de PCR, la cual fue positiva.

## Caso 8

**Figura 8:**
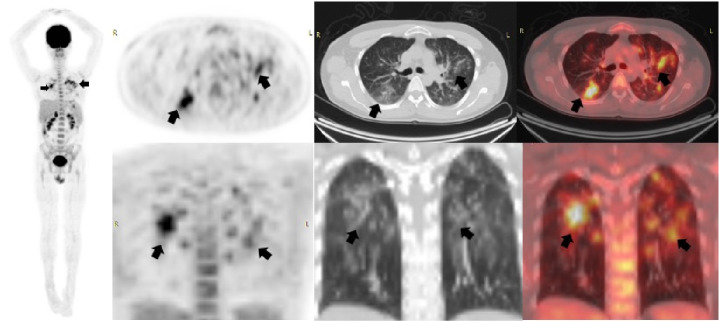
PET/CT F18-FDG en la cual se observaron múltiples opacidades nodulares y seudonodulares de varios tamaños, con incremento glucolítico y patrón de vidrio esmerilado, dispersas aleatoriamente en ambos parénquimas pulmonares con patrón multisegmentario dominante en los lóbulos superiores, incremento glucolítico dominante en el segmento anterior del lóbulo superior izquierdo y SUVmáx de 5,6, múltiples nódulos de menos de un centímetro (promedio de 5 mm), coalescentes, y opacidades en parches con patrón de vidrio esmerilado, mal definidas y bilaterales (flechas negras).

Hombre de 54 años hospitalizado con cuadro febril sin claridad sobre la causa y estudios de imágenes convencionales negativos, motivo por el cual se solicitó una PET/CT F18-FDG (figura 8) al instituto Idime. En ella se detectaron en el tórax opacidades nodulares con incremento glucolítico y apariencia de vidrio esmerilado en ambos parénquimas pulmonares. Se recomendó entonces que se le hiciera la prueba de PCR para COVID-19, la cual fue inicialmente negativa, pero que, al repetirla, arrojó un resultado positivo.

## Conclusión

Los hallazgos de las imágenes diagnósticas presentados deben servir como ayuda para que el especialista en Medicina Nuclear pueda reconocer y sospechar una eventual infección por coronavirus intercurrente en este tipo de pacientes, lo cual constituye información crucial para los familiares y el médico tratante. Además, es importante remitir al paciente para que se le haga la prueba confirmatoria y pueda instaurarse un tratamiento eficaz.
